# From Defensive Medicine to Quaternary Prevention: The Transition That Pakistan Needs

**DOI:** 10.7759/cureus.2449

**Published:** 2018-04-08

**Authors:** Samar Mahmood, Muqadus Tabraze

**Affiliations:** 1 Department of Internal Medicine, Dow University of Health Sciences (DUHS), Karachi, Pakistan

**Keywords:** defensive medicine, quaternary prevention, laboratory investigations, medical ethics, preventive medicine, overtreatment, overmedicalization, pakistan, evidence based medicine

## Abstract

The issue of overprescribing laboratory investigations is an old one in the world of medical practice and it has unfortunately seen a tremendous increase with the digitalisation of medicine, in more recent times. Phrased usually as 'defensive medicine,' this kind of overmedicalisation steers medical practice away from the ethical, skill-refining fronts on the part of doctors and imposes an unnecessary financial burden on the patients' pockets, adding to their suffering. Pakistan has not been able to save itself either, from the impropriety that roots out of what is now almost a norm in medical practice. The existent low literacy and awareness rates in the masses of the country, coupled with the cultural respect for doctors and lack of financial resources amongst the poor patients to stand up to doctors or the hospitals, have all made it even easier for physicians to get away with ordering whatever investigations they choose. The issue is a grave one and its rampancy demands that attention is drawn to it and efforts are made to transition into the practice of evidence-based medicine and quaternary prevention.

## Editorial

Today’s rapidly evolving, ‘digitalized medicine’ has created even more room for the prescription of unnecessary laboratory investigations with physicians being able to directly enter their orders into the system. The automation has eliminated the obligation upon doctors to reason out their orders with the patients first. This issue of the abuse of laboratory resources goes back a long way. In an article by Young DW in 1980, it was highlighted how, upon evaluation of the utility of laboratory tests in reaching a diagnosis in a particular clinical setting, there were only seven patients out of 80 in whom the laboratory tests proved useful. In reasoning out the logic behind such rampant over prescription, the article proposed ignorance, lack of thought, unintentional duplication of orders and mentioned a few investigations like haemoglobin concentration and biochemical profiles as those that are used for screening rather than diagnosis, just run in case, when blood is drawn for other purposes [1]. It is upsetting how, almost 40 years later, we still find ourselves surrounded by this poor practice which has only intensified in its repercussions because of the increasing cost of health care and added burden on laboratory resources.

Our country, Pakistan, has not been able to save itself either, from the impropriety that roots out of what is now almost a norm in medical practice.Baqir SM called out the exercise under ‘defensive medicine’ in his article, in 2014. He further categorized the intent behind the practice as ‘assurance’, that is in favour of the patient’s wellbeing and ‘avoidance,’ as in doctors safeguarding themselves from potential lawsuits in cases of negligence. The article pointed out how $1 of every $4 is wasted on defensive medicine and how such unnecessary testing may lead to false positive results, more invasive tests, dangerous complications, and needless hospital admissions [2].

In an emergency department evaluated for unnecessary investigations amongst their 201 admitted patients in Peshawar, Pakistan, during 2014-2015, 20.9% of the full blood counts, 14.4% random blood sugars, 2% X-rays, 6.5% electrocardiographs, and 28.9% urine analyses amongst others, were found to not have been indicated by the National Institute for Health and Care Excellence (NICE) guidelines for routine preoperative investigations, assumedly making up to 25,675 PKR (US $232) in unnecessary investigation costs and 65.7 days of additional hospital stay [3]. And this is just one healthcare center, not even from the busiest of cities in the country. Additionally, unnecessary radiation exposure as that of X-rays and computed tomography (CT) scans enhances the risk for patients developing cancers later in life, and the long interim period conveniently shifts focus from these prescribed tests as causative factors of the disease [2].

In a third world country like ours, with low literacy and awareness rates, it is even easier to get away with ordering whatever investigations the physician wills, because the cultural respect for doctors allows for ‘biomedical dominance’ to prevail. Moreover, with 29.5% people hailing from below the poverty line [4], patients find themselves lacking the authority and resources required to question or stand up to hospitals that protect themselves and their employees via their stringent policies. In a country where the average adult is said to be earning a meagre PKR 3030 (US $27) monthly [4], where only 27% enjoy full health care coverage and a significant percentage forego medical treatment completely (especially women) due to its unaffordability, it is even more inhuman to prescribe unnecessary investigations. Even in public hospitals, where the investigations and treatment are discounted/free of cost, this poor practice merely imposes an added burden on the already sparing 0.5%-0.8% of the gross domestic product (GDP) allocated to health care [5].

In addition, it is a well known, but equally well-hidden fact that certain hospitals call upon their physicians routinely, questioning them regarding their failure to generate a certain amount of revenue for their set up, thus encouraging the prescription of unnecessary investigations. Numerous private laboratories approach physicians directly as well with incentives of percentage commissions for every test prescribed. It is unfortunate how healthcare is being turned into a money minting business and the prevalence of such behavior requires some serious introspection with regards to our morality and sense of empathy.

Importantly, what does this practice say about us, as doctors? Where does the show of years of extensive studying, training and developing analytic thinking go, if we physicians are just going to abide by the lists of suggested investigations in the textbooks? Attentiveness towards patients, effective history taking, and focused physical examinations are all skills that competent doctors inculcate within themselves to obviate the need for extensive and unnecessary laboratory investigations. Physicians should be made aware of the costs of regular tests, as research has found that this cuts the patients' daily bills by as much as 27% [2]. Additionally, they should also be held accountable for all unnecessary overprescriptions, possibly by some monetary deduction from their salaries. They should be taught about good ethical practices from their early years, encouraged to develop confidence to practice evidence-based medicine, consult their colleagues regarding decision making [2] instead of counting on futile lab orders, and to read about and practice ‘quaternary prevention’, that is the act of protecting patients from overmedicalization and unnecessary medical invasions, thus improving their medical practice and reducing added patient suffering, at large.

Truly, medicine is a blend of art and science but when the science overwhelms the art, the practice of medicine becomes expensive and ineffectual.
